# The Essential Oils and Eucalyptol From *Artemisia vulgaris* L. Prevent Acetaminophen-Induced Liver Injury by Activating Nrf2–Keap1 and Enhancing APAP Clearance Through Non-Toxic Metabolic Pathway

**DOI:** 10.3389/fphar.2019.00782

**Published:** 2019-07-25

**Authors:** Zhihui Jiang, Xiao Guo, Kunpeng Zhang, Ganesh Sekaran, Baorui Cao, Qingqing Zhao, Shouquan Zhang, Gordon M. Kirby, Xiaoying Zhang

**Affiliations:** ^1^Henan Joint International Research Laboratory of Veterinary Biologics Research and Application, Anyang Institute of Technology, Anyang, China; ^2^College of Veterinary Medicine, Northwest A&F University, Xianyang, China; ^3^Department of Biotechnology, Nehru Arts and Science College, Coimbatore, India; ^4^Tangyin Administrative Office of Pharmaceutical Industry, Anyang, China; ^5^Department of Biomedical Sciences, Ontario Veterinary College, University of Guelph, Guelph, ON, Canada

**Keywords:** Artemisia vulgaris, essential oil, eucalyptol, acetaminophen, Nrf2-Keap1, liver

## Abstract

*Artemisia* has long been used in traditional medicine and as a food source for different functions in eastern Asia. *Artemisia vulgaris* L. (AV) is a species of the genus Artemisia. Essential oils (EOs) were extracted from AV by subcritical butane extraction. EO contents were detected by electronic nose and headspace solid-phase microextraction coupled with gas chromatography (HS-SPME-GC-MS). To investigate the hepatoprotective effects, mice subjected to liver injury were treated intragastrically with EOs or eucalyptol for 3 days. Acetaminophen (APAP) alone caused severe liver injury characterized by significantly increased serum AST and ALT levels, ROS and hepatic malondialdehyde (MDA), as well as liver superoxide dismutase (SOD) and catalase (CAT) depletions. EOs significantly attenuated APAP-induced liver damages. Further study confirmed that eucalyptol is an inhibitor of Keap1, the affinity *K* *_D_* of eucalyptol and Keap1 was 1.42 × 10^−5^, which increased the Nrf2 translocation from the cytoplasm into the mitochondria. The activated Nrf2 increased the mRNA expression of uridine diphosphate glucuronosyltransferases (UGTs) and sulfotransferases (SULTs), also inhibiting CYP2E1 activities. Thus, the activated Nrf2 suppressed toxic intermediate formation, promoting APAP hepatic non-toxicity, whereby APAP was metabolized into APAP-gluc and APAP-sulf. Collectively, APAP non-toxic metabolism was accelerated by eucalyptol in protecting the liver against APAP-induced injury, indicating eucalyptol or EOs from AV potentials as a natural source of hepatoprotective agent.

**Figure:**
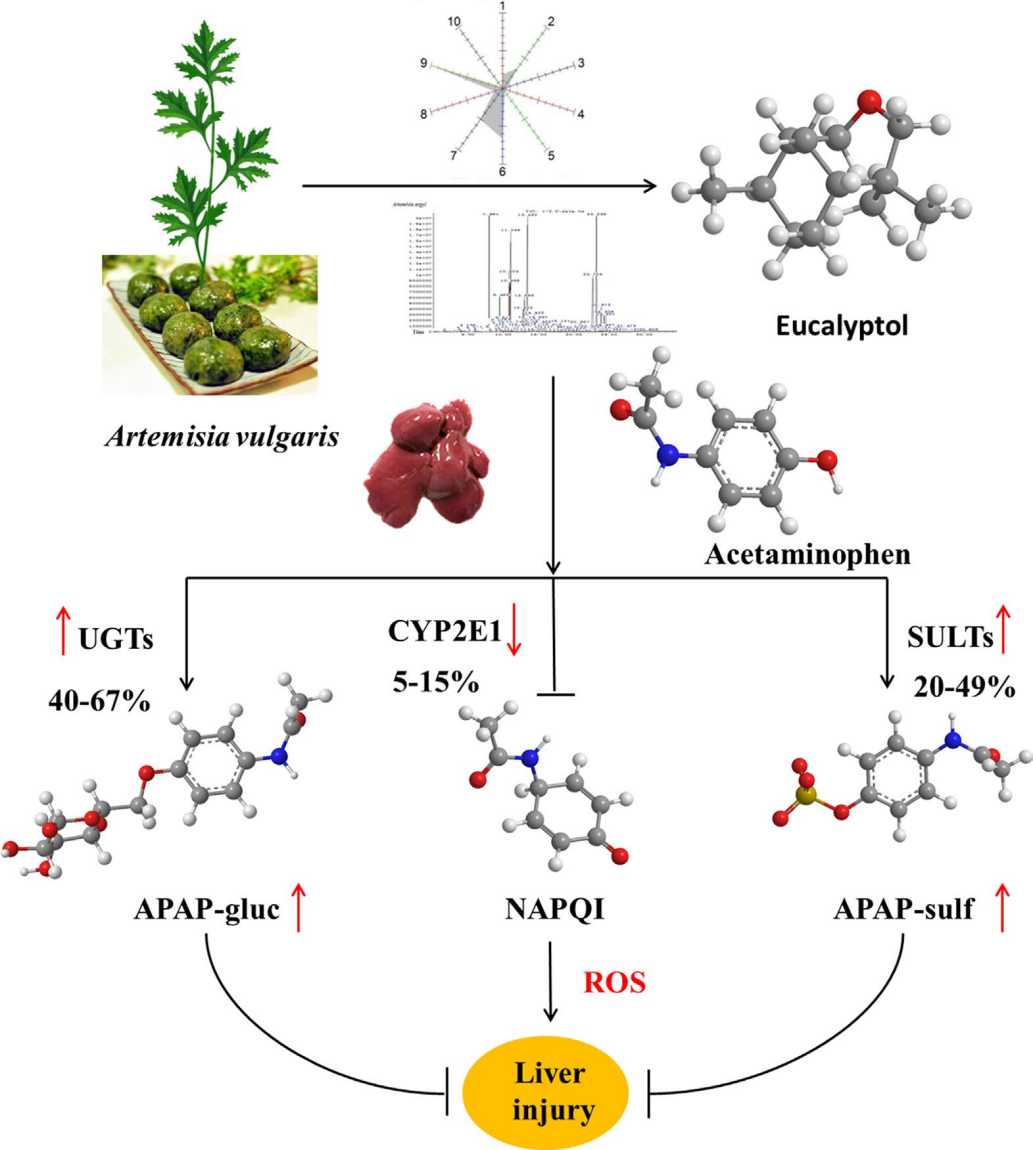
Graphical Abstract

## Introduction

*Artemisia* is a class of fragrant annual herb species of the composite family, distributed widely in Asia, Europe, and North America. It has a long history of traditional and popular use as both medicine and food with medicinal literature documentation since the Eastern Han Dynasty in 1st-century China (Song et al., 2016). *Artemisia* leaves have been considered to have a broad range of functions including anti-diarrhea, anti-inflammation, cough relief, antioxidant, and hepatoprotection (Giangaspero et al., 2009; Ferreira and Luthria, 2010; Obolskiy et al., 2011; Meng et al., 2018). The search for active compounds from Artemisia has led to the discovery and isolation of many phytochemicals and essential oils (EOs) with interesting activity. Artemisinin, a sesquiterpene lactone with antimalarial properties, is a prominent example (Gaur et al., 2014). EOs from other plants have been used in the treatment of inflammation, against free radicals, and for their hepatoprotective effect (Yoon et al., 2010; Younsi et al., 2017). *Artemisia vulgaris* L. (AV) is a major and common *Artemisia* plant that was first recorded by Ben-Cao-Gang-Mu (Ming Dynasty, 16th century by Shizhen Li), who stated that the leaves should be collected and dried in summer for medical uses, including improving Yang-qi and decreasing skeleton raw.

Eucalyptol (1,8-cineole) is one of the major essential oils in AV. Eucalyptol has been used as a percutaneous penetration enhancer (Levison et al., 1994), an antibacterial and expectorant (Giamakis et al., 2001), and as an anti-inflammatory (Juergens et al., 1998) or antihypertensive agent (Lahlou et al., 2002). Eucalyptol acted as a strong inhibitor of proinflammatory cytokines such as tumor necrosis factor (TNF)-α and interleukin (IL)-1β and showed an analgesic effect in an inflammatory model. Even though there is a report about eucalyptol acting against fatty liver in mammals and zebrafish (Cho, 2012; Murata et al., 2015), the effect and mechanism of eucalyptol against drug-induced liver injury remain unclear.

Drug-induced liver injury has become a major public health concern (Asrani et al., 2018; Real et al., 2019). Acetaminophen (APAP) overdose is the leading cause of drug-induced acute liver failure. Oxidative stress is considered to be the primary cellular event in APAP-induced liver injury (Nikravesh et al., 2018; Zhao et al., 2018). Under overdose conditions, most APAP is metabolized by phase II conjugating enzymes, mainly sulfotransferase (SULT) and UDP-glucuronosyltransferase (UGT), converting it to nontoxic compounds, which are then excreted with the urine. The remaining APAP, approximately 5–9%, is metabolized by the cytochrome P450 enzymes (CYPs), mainly CYP2E1, into the highly reactive intermediate metabolite N-acetyl-p-benzoquinone imine (NAPQI). NAPQI is usually rapidly detoxified by conjugating it with glutathione (GSH). However, when phase II metabolizing enzymes are saturated after APAP overdose, excessive NAPQ1 can deplete GSH, leading to covalent binding of sulfhydryl groups in cellular proteins and resulting in liver oxidative stress (Lancaster et al., 2015; Du et al., 2016). Nuclear factor erythroid 2-related factor 2 (Nrf2) is likely activated by redox status changes induced by NAPQI. Nrf2 dissociates from Keap1 and translocates into the nucleus to stimulate transcription of target genes with the help of small Maf proteins. These preceding processes led to the transcriptional activation of antioxidant enzymes, such as NAD(P)H, quinone oxidoreductase 1 (NQO1), heme oxygense-1 (HO-1), glutamate cysteine ligase (GCL), and glutathione S-transferase A (GSTA), increasing the expression of SOD and CAT (Loboda et al., 2016). In this study, the common reagent APAP that induced drug liver injury was chosen to explain the mechanism of AV and eucalyptol hepatoprotection.

## Materials and Methods

### Phylogenetic Analysis

The plant sample 2 (*Artemisia vulgaris*) was collected in Tangyin, Henan province (N 38°39′27.97″, E 104°04′58.66″) with monsoon climate of medium latitudes and cinnamon soil. According to plant morphological analysis, the collected sample had nine vein stem leaves compared with sample 1, *Artemisia argyi* (five vein stem) cultivated in Hubei province (N30°13′39.89″, E115°26′10.36″). For both samples, single leaves were collected. Genomic DNA was extracted using the modified hexadecyl trimethyl ammonium Bromide (CTAB) method and diluted to a concentration of 20 ng/μl in ddH_2_O. The PCR primers ITS2 are S2F: ATGCGATACTTGGTGTGAAT, S3R: GACGCTTCTCCAGACTACAAT. PCR reactions (25 µl) were composed of 4 μl genomic DNA (20 ng/μl), 2.5 μl PCR buffer (10×), 2.0 μl dNTPs (2 mmol), 2.0 μl MgSO_4_ (25 mmol), primers (2 μmol), 0.4 U Kod-Plus-Neo DNA polymerase (TOYOBO, Osaka, Japan), and sterile water.

PCR amplification started with the denaturing step at 94°C for 3 min, followed by 36 cycles of denaturation at 98°C for 20 s, annealing at 58–60°C for 20 s, extension at 68°C for 50 s, and a final extension at 68°C for 6–8 min before cooling to 10°C.

The original sequences were assembled using CodonCode Aligner V3.0 (CodonCode Co., Centerville, MA, USA). The ITS2 sequences were subjected to Hidden Markov Model (HMM) (Keller et al., 2009) model analysis to remove the conserved 5.8S and 28S DNA sequences (Koetschan et al., 2012). The ITS2 sequences were aligned using Clustal W (Thompson et al., 2002), and the genetic distances were computed using MEGA 6.0 according to the Kimura 2-Parameter (K2P) model (Tamura et al., 2011). Subsequently, MEGA6.0 software 20 was employed to construct an unrooted phylogenetic tree based on alignments using the neighbor-joining (NJ) method with the following parameters: JTT model, pairwise gap deletion, and 1,000 bootstraps. Furthermore, maximum likelihood, minimal evolution, and PhyML methods were also applied in the tree construction to validate the results from the NJ method. Annotated ITS2 sequences of Sample 1, Sample 2, Artemisia vulgaris, Artemisia lavandulifolia, Artemisia argyi, and Artemisia annua were folded by energy minimization using the ITS2 database web server (http://its2.bioapps.biozentrum.uni-wuerzburg.de/) for secondary structure analysis.

### Subcritical Butane Extraction of AV-EOs and Purification

The EOs were obtained by subcritical butane extraction apparatus (Henan Subcritical Biological Technology Co., Ltd., Anyang, China). The liquid/solid ratio was 30:1, the temperature was 45°C, extraction time was 34 min, and the particle size was 0.26 mm. The extract was subjected to hydrodistillation in Clevenger-type apparatus for 2 h. The oil/water emulsion produced was collected and stored at 4°C overnight to separate the essential oil from the residual water. The essential oil was then removed and stored in an amber glass bottle at room temperature until further use.

### Electronic Nose Analysis

The constituent of EOs from AV was captured by measuring the headspace PEN3 (Airsense Analytics system GmbH, Schwerin, Germany). The e-nose system consisted of a fully automated Headspace-Sampler, an array of ten sensors (Alpha MOS company, France), and an electronic unit for data acquisition. The MOS sensors consisted of W1C (aromatic), W5S (broadrange), W3C (aromatic), W6S (hydrogen), W5C (arom-aliph), W1S (broad-methane), W1W (sulphur-organic), W2S (broad-alcohol), W2W (sulph-chlor), and W3S (methane-aliph). Volatile organic compounds (VOCs) were injected into the Portable Electronic Nose PEN3 system using an auto-sampler at a low rate of 60 ml/min from 10 ml sealed glass vials containing 1 ml of oil sample. The VOCs were carried by pure gas (carrier gas) at 5 psi and exposed to the sensor chambers. The relative change in the resistance (G0/G) value determines the response of sensors for oil samples. TheΔ*R/R* value was monitored precisely for 130 s. The data were analyzed using Loading (Lo) and principal component analysis (PCA). The PCA values were determined using Eq 1

(Eq 1) $$r_{\mathrm{ij}}=\frac{\overset{n}{\underset{k=1}{\sum }}(x_{\mathrm{ki}}-{\bar{x}}_{j})}{\sqrt{\overset{n}{\underset{k=1}{\sum }}{\left(x_{\mathrm{ki}}-{\bar{x}}_{j}\right)}^{2}\times }\sqrt{\overset{n}{\underset{k=1}{\sum }}(x_{\mathrm{kj}}-{\bar{x}}_{j})}}(\mathrm{ij})=1,2,\cdots ,p)$$

### Determination of Essential Oil Ingredients by Headspace Solid-Phase Microextraction Coupled to Gas Chromatography (HS-SPME-GC-MS)

The EOs were analyzed on an HS-SPME-GC-MS system consisting of commercial manual sampling SPME devices (Supelco, Inc. Bellefonte, PA, USA). SPME ﬁbers with 100 μm polydimethylsiloxane (PDMS), 65 μm PDMS/divinylbenzene (PDMS/DVB), 85 μm polyacrylate (PA), 85 μm carboxen/PDMS (CAR/PDMS), and 70 μm carbowax/DVB (CW/DVB) were used. The analysis was carried out on a GC system (GC-2010, Shimadazu Tokyo, Japan) coupled with a ﬂame ionization detector (FID). All the ﬁbers were conditioned before use in the GC injector, according to the instructions provided by the manufacturer. Separation was performed using a DB-5 capillary column (30 m × 0.25 mm I.D. and 0.25 μm, J&W Scientiﬁc, CA, USA).

The instrument parameters for the analysis were as follows: N2 ﬂow: 1.47 ml/min; column temperature program: held at 40°C for 3 min, then increased from 40 to 70°C at 15°C/min and maintained for 1 min, and then increased to 250°C at 30°C/min and held for 1 min. The detector temperature was held at 280°C. In optimized conditions, the temperature of the injector was set at 250°C, and the desorption process was performed in the splitless mode for 2 min.

For the HS-SPME experiments, EOs (3 ml) were placed in a 10 ml glass vial. The vial was closed with Teﬂon-lined septa (Supelco, Pennsylvania, USA). The standard solutions and EOs samples were stored at room temperature. Afterwards, a ﬁber was introduced into the headspace of the vial for 10 min at the same extraction temperature. After extraction, the ﬁber was removed from the vial, inserted into the inlet of the GC, and desorbed at 250°C for 2 min.

### Compounds, Targets, and Pathway Analysis

AV compounds (85) were used to analyze the ADME properties using SwissADME (swissadme.ch/index.php). Based on the Lipinski rule, a total of 31 compounds were finalized for target fishing and pathway analysis. All selected compounds from SN structural data were retrieved from Pubchem (https://pubchem.ncbi.nlm.nih.gov/). Active components were identified and compared with Similarity Ensemble Approach (SEA) (http://sea.bkslab.org/) (Keiser et al., 2007) and Drug Repositioning and Adverse Reaction *via* Chemical-Protein Interactome (DRAR-CPI) (http://cpi.bio-x.cn/drar/) (Luo et al., 2011). Furthermore, Comparative Toxicogenomics Database (CTD) (http://ctdbase.org/) was applied for the target mining process. The target genes of AV were applied for pathway analysis using The Database for Annotation, Visualization and Integrated Discovery 6.8 server (DAVID) (http://david.abcc.ncifcrf.gov): analytical tools used to identify the gene or proteins systematically. KEGG pathway information was retrieved (**Supplementary Table 1**) (Dennis et al., 2003).

### Experimental Animals

The experimental protocol was reviewed and approved by the Ethics Committee of the Institute of Modern Biotechnology for the Use of Laboratory Animals. A total of 42 (15 male and 15 female) inbred Kunming mice (18–20 g) aged 4 weeks and 12 inbred SD rats (200–220 g) were obtained from the animal center of Zhengzhou University (Zhengzhou, China). The animals were kept under controlled conditions at temperature 22 ± 2°C, humidity 70% ± 4% with 12 h light–dark cycling.

### Animal Treatments

*Experiment 1:* Mice were randomly divided into five groups with six mice in each group (n = 6, three males and three females). Control group: Tween-80 (100 ml/kg); APAP group: 300 mg/kg; APAP-Essential oil (APAP-EO) group: essential oil was emulsified in Tween-80 (95 %), essential oil (100 ml/kg) with oral administration after APAP (300 mg/kg) treatment for 1 h; APAP-eucalyptol (APAP-EU) group: eucalyptol (5 ml/kg) with oral administration after APAP (300 mg/kg) treatment for 1 h; Essential oil group (EO): 5% essential oil (100 ml/kg); eucalyptol (EU) group: 5 ml/kg; Positive group: NAC (100 mg/kg) with oral administration after APAP treatment for 1 h.

*Experiment 2:* Rats were randomly divided into two groups of five each. APAP group: 300 mg/kg; eucalyptol-APAP group: 5 ml/kg eucalyptol with oral administration after APAP (300 mg/kg) treatment for 1 h. After eucalyptol treatment for 0.2, 0.5, 0.75, 1, 1.5, 2, 4, and 6 h, the plasma was collected for high performance liquid chromatography (HPLC) or high performance liquid chromatography-tandem mass spectrometry (HPLC-MS/MS) analysis. The plasma samples were mixed with acetonitrile and centrifuged at 13,000 g for 15 min at 4°C.

### Determination of Serum ALT and AST Levels

Enzymatic activities of aspartate aminotransferase (AST) and alanine aminotransferase (ALT) in serum were evaluated by spectrophotometer using commercial diagnostic kits (Nanjing Jiancheng Institute of Biotechnology, Nanjing, China).

### Determination of Histology

Liver tissues were fixed in 10% formalin and embedded in paraffin for histological assessment. Samples were sectioned 5 µm and stained with hematoxylin and eosin. The slides were examined under a light microscope with photo-micrographic attachment.

### Determination of Hepatic ROS, SOD, CAT, and MDA Levels

Frozen liver tissues were homogenized in ice-cold PBS. The supernatants were collected after the homogenate was centrifuged at 3000 g, 4°C for 10 min. Superoxide dismutase (SOD), catalase (CAT), and malondialdehyde (MDA) levels were measured with a spectrophotometer using the commercially available assay kits as per the manufacturer’s instructions (Nanjing Jiancheng Bioengineering Institute, Nanjing, China). The reactive oxygen species (ROS) levels were assayed with a fluorescence detector using commercial kits (Jiancheng Bioengineering Institute, Nanjing, China). The protein concentrations in tissue homogenates were measured with Bradford protein assay using bovine serum albumin as the standard (Jiancheng Bioengineering Institute, Nanjing, China).

### Determination of Drug Metabolism-Related Gene Expressions

Total mRNA was isolated from frozen liver tissues using a Total RNA kit (Tiangen, Beijing, China). Quantitative real-time PCR (qPCR) was carried out for the amplification of cDNA using 2×SYBR Green I PCR Master Mix (Vazyme, Nanjing, China). The PCR procedure consisted of 95°C for 30 s followed by 35 cycles of 95°C for 15 s, 58°C for 30 s, and 72°C for 30 s. The PCR primers were used as shown in **Table 1**. The melting curve and dissociation curve were extrapolated to confirm primer specificity and product purity. The relative abundance of each mRNA was calculated with the formula 2^−(ခΔΔCt)^, where ΔΔCt = (Ct_Target_ – Ct_GAPDH_) treatment − (Ct_Target_ − Ct_GAPDH_) control.

**Table 1:** Primers used for quantitative real-time PCR.

Target gene	Sense 5’-3’	Antisense 5’-3’
Nrf2	GCTGATGGAGTACCCTGAGGCTAT	ATGTCCGCAATGGAGGAGAAGTCT
HO-1	TGCCAGTGCCACCAAGTTCAAG	TGTTGAGCAGGAACGCAGTCTTG
NQO1	GGAGACAGCCTCTTACTTGCCAAG	CCAGCCGTCAGCTATTGTGGATAC
GCLC	TGAGATTTAAGC CCCCTCCT	TTGGGATCAGTCCAGGAAAC
GSTA2	TCAGTAACCTGCCCACAGTGAAG	GCATGTTCTTGACCTCTATGGCTGG
UGT1A1	CACGCTGGGAGGCTGTTAGT	CACAGTGGGCACAGTCAGGTA
UGT1A6	CACGTGCTACCTAGAGGCACAG	GACCACCAGCAGCTTGTCAC
UGT1A9	GAAGAACATGCATTTTGCTCCT	CTGGGCTAAAGAGGTCTGTCATAGTC
SULT1A1	CCCGTCTATGCCCGGATAC	GGGCTGGTGTCTCTTTCAGAGT
SULT2A1	TAGGGAAAAATTTAGGGCCAGAT	TTGTTTTCTTTCATGGCTTGGA
CYP2E1	CACCGTTGCCTTGCTTGTCTG	CTCATGAGCTCCAGACACTTC
GAPDH	ACATGGCCTCCAAGGAGTAAGA	GATCGAGT TGGGGCTGTGACT

### Determination of CYP2E1 and Nrf2 Protein Expression

For Nrf2 expression analysis, the extraction and isolation of cytoplasmic and nuclear proteins were performed using a Cytoplasmic and Nuclear Protein Extraction Kit (Beyotime, Nanjing, China), according to the manufacturer’s instructions. For CYP2E1 expression analysis, the extraction and isolation of microsomal proteins were carried out as described previously (Jiang et al., 2016; Chen et al., 2019). The protein concentration was determined by BCA assay kit (Beyotime, Nanjing, China). Equal amounts of protein extracts were subjected to SDS–polyacrylamide gel electrophoresis under reducing conditions in concentrate protein gel 5% (pH = 6.8) and separating protein gel 12% (pH = 8.8). The separated proteins were transferred to PVDF membranes using tank transfer for 2 h at 200 mA in Tris–glycine buffer with 15% methanol. Membranes were blocked with 5% skimmed milk for 3 h and incubated for 12 h with anti-CYP2E1 (1:1500, Boster, Wuhan, China), anti-Nrf-2 (1:500, Bioss, Beijing, China), anti-GAPDH (1:1000, Boster, Wuhan, China), and anti-Lamin B (1:500, Bioss, Beijing, China) for 2 h at 37°C. The secondary antibodies (IgG/HRP) were incubated for 2 h at 37°C. The images of the blots were visualized by ECL (Genshare, Xi’an, China).

### Molecular Docking

Molecular docking was employed to study the interactions between the eucalyptol and the Keap1 using Autodock vina version 1.1.2 package. The three-dimensional (3D) structure of the Keap1 (PDB ID: 3WN7) was retrieved from the RCSB Protein Data Bank (http://www.rcsb.org). The 2D structure of the eucalyptol was drawn by ChemBioDraw Ultra 14.0 and converted to the 3D structure by ChemBio3D Ultra 14.0 package. The AutoDockTools version 1.5.6 was used to obtain the docking input files. The binding site of the Keap1 was identified as center_x: 3.766, center_y: 1.122, and center_z: 19.296 with dimensions size_x: 15, size_y: 15, and size_z: 15. To increase the accuracy of the calculation, the value of exhaustiveness was set to 20. In addition, the default parameters were used, if it was not mentioned. The best docking pose as judged by the Vina score was chosen and further analyzed using PyMoL 1.7.6 software (http://www.pymol.org/).

### SPR Interaction and Affinity Analysis

To understand the interactions between eucalyptol and the Keap1 protein, we performed an affinity measurement using surface plasmon resonance (SPR) technology. The SPR validation experiment was performed with the bScreen LB 991 Label-free Microarray System (Berthold Technologies, Germany). To validate detection of the eucalyptol–Keap1 interactions, the photo-cross-linker sensor chip was used. Rapamycin and DMSO were selected as system positive control and negative control, respectively. We arranged kinetic constant tests with FKBP12 immediately after the sample tests. During the SPR test, the Keap1 protein (MyBioSource, Vancouver, Canada) was diluted separately with running buffer to 200, 400, 800, 1600, and 3200 nM and injected for 600 s at a flow rate of 0.5 µl s^−1^ at associating stage, followed by running buffer for 360 s at a flow rate of 0.5 µl s^−1^ at each dissociating stage. At the end of each associating–dissociating cycle, the surface was regenerated to remove any remaining bound material with a pulse of 10 mM glycine–HCl (pH 2.0) at 20 µl min^−1^ for 30 s.

The raw sensorgrams and measurements of the binding process of ligands and proteins were recorded in real time. The response unit (RU) of surface resonance was compared to determine the different binding affinities between each sample dot. The response unit data collected on the SPR biosensor was further processed to eliminate any artifacts such as non-specific binding and differences in buffer composition. The process and analysis of association and dissociation rate constants (*K* *_a_*/ *K* *_on_* and K_d_/K_off_ respectively) and the equilibrium dissociation constant (*K* *_D_* *, K* *_d_*/ *K* *_a_*) were performed using the data analysis software of the bScreen LB 991 unlabeled microarray system according to a single-site binding model (1:1 Langmuir binding) with mass transfer limitations for binding kinetics determination.

### LC-MS/MS Analysis of APAP and its Metabolites

Plasma samples were obtained 0.2, 0.5, 0.75, 1, 1.5, 2, 4, and 6 h after different treatments. The concentrations of APAP and its conjugated metabolites were analyzed using modified LC-MS/MS. Briefly, plasma samples were centrifuged at 13,000g for 15 min at 4°C. The supernatants were diluted with ultrapure water. Reversed phase chromatography of APAP, APAP-glucuronide (APAP-gluc), and APAP-sulfate (APAP-sulf) was carried out using an ACE AQ C18 column (Advanced Chromatography Technologies, UK; 2.1×100 mm, 3 μm) at 50°C at a flow rate 0.3 ml/min. Mobile phase A was 0.2% formic acid in acetonitrile, while mobile phase B was 0.1% formic acid in methanol. The condition of chromatographic separation was 2% B from 0 to 0.5 min, held at 95% B for 0.8 min and then to 2% B at 3.31 min, followed by 5 min of column equilibration using 2% B.

The mass spectrometer was operated in ESI+Agilent Jet Stream mode with multiple reaction monitoring (MRM). The target compounds were detected by monitoring the m/z transition: m/z 150.0→107.0 for APAP, m/z 326.0→150.0 for APAP-gluc, and m/z 230.0→150.0 for APAP-sulf, with a dwell time of 100 ms for each mass transition. TIS temperature was 500°C, and TIS voltage was 3.5 kV. Curtain gas, nebulizing gas, TIS gas, and collision gas was 25, 90, 80, and 10 units, respectively. Collision energy and collision cell exit potential are 14 and 80 V for APAP, 5 V and 100 V for APAP-gluc, and 30 and 110 V for APAP-sulf, respectively. The mass spectrometer was operated at unit mass resolution for both Q1 and Q3 quadruples.

The standard curve consisted of samples containing 0, 20, 40, 80, 160, and 320 µg/ml of APAP, APAP-gluc, and APAP-sulf.

### Statistical Analysis

The results were presented by means of, at least, five measurements, duplicated for each set, having a coefficient of variation less than 5%. One-way ANOVA followed by Duncan’s multiple range test (*p* < 0.05) with SPSS 20.0 (SPSS Inc., Chicago, IL, USA) was applied for the mean values compared.

## Results

### Phylogenetic Relationships

**Figure 1:**
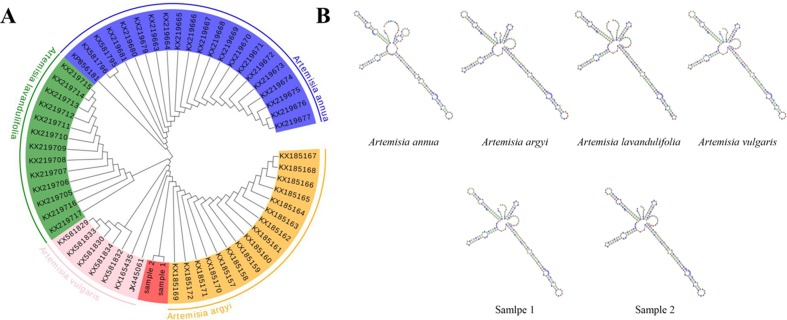
Species identification of sample 2 by phylogenetic tree and ITS2 secondary structure. **(A)** Phylogenetic tree of the four Artemisia species constructed with the ITS2 sequences using the neighbor-joining (NJ) method; **(B)** ITS2 secondary structure.

DNA barcode analysis with ITS2 sequence was used to investigate the evolutionary history and phylogenetic relationships of sample 1. A phylogenetic tree based on NJ cluster algorithm was constructed in **Figure 1A**. NJ sequence similarity analysis discovered that sample 1 was close to its close species from Artemisia (*Artemisia vulgaris*, *Artemisia lavandulifolia* and *Artemisia annua*). The branches indicate the bootstrap values for 1000 replicates. Furthermore, inter-species variations were also calculated and the results showed that Sample 1 and Artemisia argyi showed the highest similarity (0.005), while Sample 2 and Artemisia vulgaris showed the highest similarity (0.003) as presented in **S. Table 2**. The secondary structure of ITS2 of Sample 1 is similar to *Artemisia argyi*, while Sample 2 is similar to *Artemisia vulgaris* (**Figure 1B**). Compared with multiple analysis methods, the results show that Sample 1 was *Artemisia argyi* L, and Sample 2 was *Artemisia vulgaris* L.

### E-Nose Analysis

**Figure 2:**
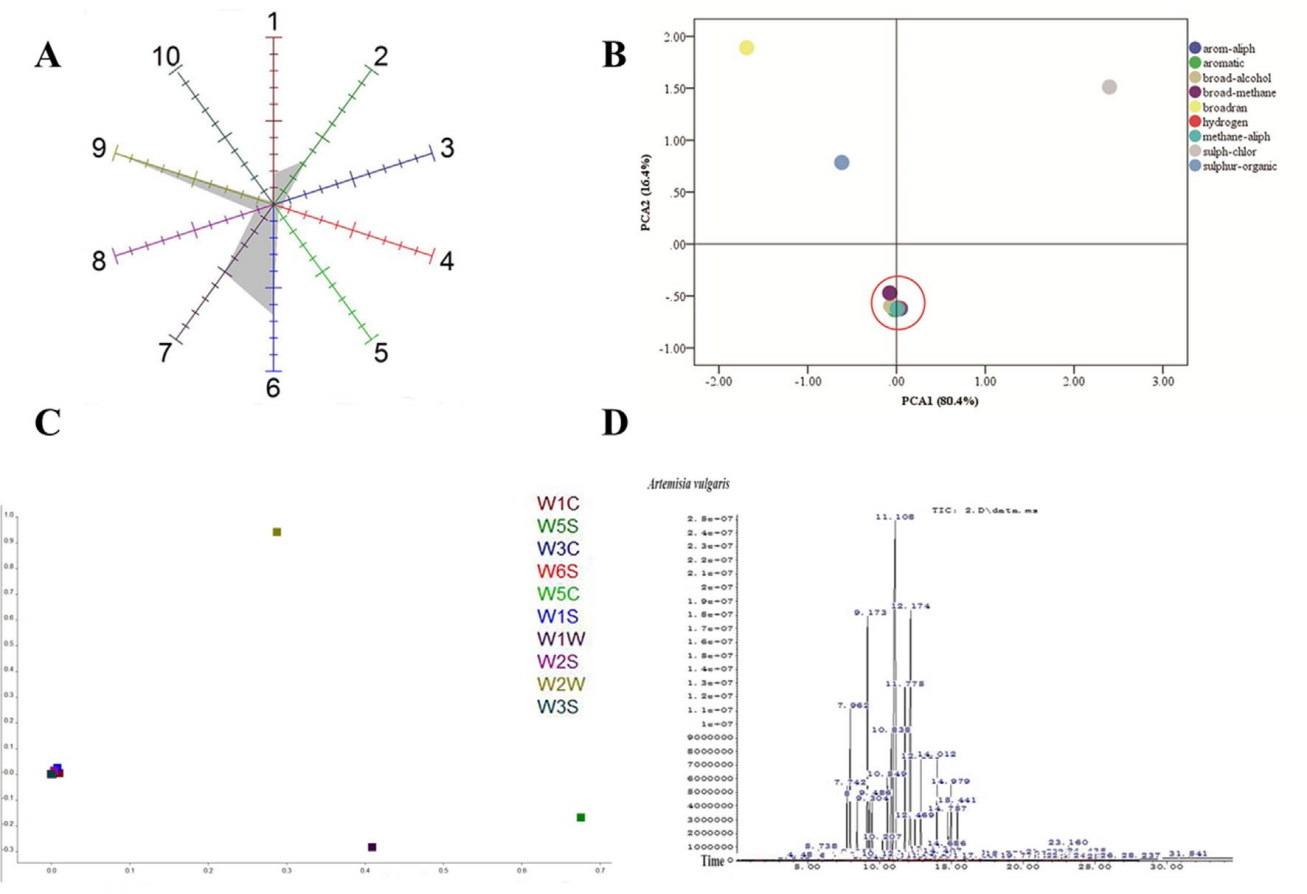
Component analysis of *Artemisia vulgaris* L. essential oils (AV-EOs). **(A)** Polar plot display of sensor values of EOs from AV at 57 s given by the cursor in the measurement window; **(B)** principal component analysis (PCA); **(C)** loading (Lo) analysis; W1C (aromatic), W5S (broadrange), W3C (aromatic), W6S (hydrogen), W5C (arom-aliph), W1S (broad-methane), W1W (sulphur-organic), W2S (broad-alcohol), W2W (sulph-chlor), W3S (methane-aliph); **(D)** the headspace solid-phase microextraction coupled with gas chromatography (HS-SPME-GC-MS) diagram of AV.

To analyze the volatile organic compounds from AV, the e-nose was used. The polar plot display of sensor values of EOs from AV at 57s was given by the cursor in the measurement window. The sensor of W2W, W1W, and W1S was highly sensitive to the EOs of AV **(** **Figure 2A** **)**, indicating that the collected EOs contained sulph-chlor, sulphur-organic and broad-methane. The flying data were examined with PCA to visualize the response patterns in the feature space of principal components (PC). Two PCs, namely, PC1 and PC2, which explained 80.4% and 16.4%, respectively, of the data variance, were chosen based on the Eigen values (*p* > 0.5). Using PCA, the three main odors of AV were sulph-chlor, sulphur-organic, and broadrange **(** **Figure 2B** **)**. The Lo analysis revealed that W5S and W1W have higher response to the compounds of AV, indicating that the broad range and sulphur-organic were present in EOs of AV. W6S, W5C, W2S, and W3S were not sensitive to the EOs of AV **(** **Figure 2C** **)**.

### Phytochemical Analysis of AV-EOs

Eighty components were identified from EOs of AV by HS-SPME-GC-MS analysis **(** **Table 2**, **Figure 2D** **)**. The three highest components of AV were eucalyptol (28.07%), Cis-β-Terpineol (16.44%), Byciclo [3.1.0] hex-2-ene, 4-methyl-1-(1-methylethyl) (8.89%), Benzene,1-methyl-4-(1-methylethyl) (7.38%), 1,4-Cyclohexadiene, 1-methyl-4-(1-methylethyl) (4.88%), 1S-а-Pinene (4.16%), and 3-Cyclohexen-1-ol, 4-methyl-1-(1-methylethyl) (3.35%).

**Table 2:** The compositions of *Artemisia vulgaris* L. (AV).

*Artemisia vulgaris*					
Rank	Time (min)	Compound	CAS#	Matching rate	Relative amount
1	1.423	1,3-Dioxolane-4-methanol	005464-28-8	64	0.03%
2	1.623	Oxirane,(propoxymethyl)-	003126-95-2	64	0.02%
3	1.805	3(2H)-Furanone,dihydor-2-methyl-	003188-00-9	42	0.00%
4	1.846	Methacrolein	000078-85-3	72	0.01%
5	2.382	2-Butanone,3-methyl-	000563-80-4	49	0.02%
6	2.452	Oxirane,tirmethyl-	005076-19-7	43	0.00%
7	2.799	1,3-Dioxolane,4-methyl-	001072-47-5	47	0.01%
8	3.346	1-Butanol,3-methyl-	000123-51-3	59	0.02%
9	3.393	1-Heptene	000592-76-7	50	0.04%
10	3.846	Toluene	000108-88-3	70	0.02%
11	4.276	1-Octene	000111-66-0	94	0.07%
12	4.481	Hexanal	000066-25-1	90	0.07%
13	5.74	2-Hexanal	000505-57-7	96	0.42%
14	6.74	1-Nonene	000124-11-8	95	0.05%
15	7.622	Tircyclo[2.2.1.0(2,6)]heptane,1,7,7-trimethyl-	000508-32-7	96	0.07%
16	7.74	Bicyclo[3.1.0]hexane,4-methyl-1-(1-methylethyl)-,didehydro derive.	058037-87-9	94	2.01%
17	7.964	1S-.alpha.-Pinene	007785-26-4	96	4.16%
18	8.264	Bicyclo[3.1.0]hex-2-ene,4-methylene-1-(1-methylethyl)-	036262-09-6	91	0.13%
19	8.466	Camphene	000079-92-5	97	1.74%
20	8.558	1-Butanol,4-(phenylmethoxy)-	004541-14-4	47	0.03%
21	8.852	Benzaldehyde	000100-52-7	97	0.11%
22	9.046	Benzaldehyde	000100-52-7	42	0.02%
23	9.175	Byciclo[3.1.0]hex-2-ene,4-methyl-1-(1-methylethyl)-	028634-89-1	91	8.89%
24	9.305	.beta.-Pinene	000127-91-3	97	1.54%
25	9.487	1-Octen-3-ol	003391-86-4	90	2.65%
26	9.675	1-Octen-3-ol	003391-86-4	43	0.10%
27	9.734	2-Propyl-1-pentanol	058175-57-8	50	0.23%
28	10.122	Octanal	000124-13-0	80	0.10%
29	10.205	.alpha.-Phellandrene	000099-83-2	90	0.75%
30	10.546	(+)-4-Carene	029050-33-7	98	2.92%
31	10.84	Benzene,1-methyl-4-(1-methylethyl)-	000099-87-6	97	7.38%
32	11.11	Eucalyptol	000470-82-6	97	28.07%
33	11.775	1,4-Cyclohexadiene,1-methyl-4-(1-methylethyl)-	000099-85-4	94	4.88%
34	12.175	Cis-.beta.-Terpineol	007299-40-3	96	16.44%
35	12.469	(+)-4-Carene	029050-33-7	98	1.04%
36	12.599	Benzene,1-methyl-4-(1-methylethenyl)-	001195-32-0	94	0.16%
37	12.805	Acetoacetic acid isoamyl ester	002308-18-1	43	0.03%
38	13.116	Terpineol, Cis-.beta.-	007299-41-4	90	0.06%
39	13.246	1-Methoxy-1,3-cyclohexadiene	002161-90-2	60	0.03%
40	13.328	1,6-Dimethylhepta-1,3,5-triene	1000196-61-0	94	0.16%
41	13.446	2-Cyclohexen-1-ol,1-methyl-4-(1-methylethyl)-,trans-	029803-81-4	96	0.30%
42	13.522	Cyclohexanone,5-methyl-2-(1-methylethenyl)-,trans-	029606-79-9	78	0.07%
43	13.599	Cyclopentasiloxane,decamethyl-	000541-02-6	80	0.02%
44	13.904	2-Cyclohexen-1-ol,1-methyl-4-(1-methylethyl)-,cis-	029803-82-5	46	0.14%
45	14.01	Camphor	000076-22-2	98	3.02%
46	14.193	Benzyl alcohol	000100-51-6	55	0.15%
47	14.275	2,6-Dimethylbicyclo[3.2.1]octane	000215-28-2	72	0.13%
48	14.404	Bicyclo[2.2.1]heptan-3-one,6,6-dimethyl-2-methylene-	1016812-40-1	97	0.16%
49	14.687	3-Cyclohexene-1-methanol,.alpha.,.alpha.,4-trimethyl-,(S)-	010482-56-1	72	0.39%
50	14.757	Borneol	010385-78-1	97	1.87%
51	14.981	3-Cyclohexen-1-ol,4-methyl-1-(1-methylethyl)-	000562-74-3	93	3.35%
52	15.44	3-Cyclohexene-1-methanol,.alpha.,.alpha.,4-trimethyl-,(S)-	010482-56-1	87	2.97%
53	16.504	Bicyclo[2.2.1]heptan-2-ol,1,7,7-trimethyl-,acetate,(1S-endo)-	005655-61-8	72	0.11%
54	16.934	Cyclohexene,1-methyl-3-(1-methylethenyl)-,(.+/-.)-	000499-03-6	91	0.06%
55	17.087	2-Cyclohexen-1-one,2-methyl-5-(1-methylethenyl)-,(S)-	002244-16-8	38	0.06%
56	18.428	1-Cyclohexene-1-carboxaldehyde,4-(1-methylethenyl)-	002111-75-3	98	0.25%
57	18.751	Bornyl acetate	000076-49-3	99	0.15%
58	19.604	Cyclohexasiloxane,dodecamethyl-	000540-97-6	91	0.02%
59	19.781	Triallylmethylsilane	001112-91-0	38	0.07%
60	21.557	Phenol,2-methoxy-3-(2-propenyl)	001941-12-4	98	0.45%
61	22.145	Copaene	003856-25-5	98	0.12%
62	22.351	.alpha.-Bourbonene	1000293-01-9	87	0.04%
63	22.574	Benzenepropanoic aid,10-undecenyl	000281-79-0	43	0.03%
64	22.645	Phenol,4-methyl-	1000106-44-5	64	0.04%
65	22.757	Tetradecane	000629-59-4	97	0.04%
66	23.027	3-Carene	013466-78-9	81	0.04%
67	23.157	Caryophyllene	000087-44-5	99	0.48%
68	23.421	Bicyclo[3.1.1]hept-2-ene,2,6-dimethyl-6-(4-methyl-3-pentenyl)-	017699-05-7	98	0.04%
69	23.786	1,6,10-Dodecatriene,7,11-dimethyl- 3-methylene-,(Z)-	028973-97-9	93	0.11%
70	23.863	.alpha.-Caryophyllene	006753-98-6	97	0.05%
71	23.986	1H-Benzocycloheptene,2,4a,5,6,7,8,9,9a,-octahydro-3,5,5-trimethyl-9-methylene-,(4aS-cis)-	003853-83-6	70	0.03%
72	24.168	Naphthalene,decahydro-4a-methyl-1-methylene-7-(1-methylethylidene)-,(4aR-trans)-	000515-17-3	98	0.05%
73	24.333	1,6,10-Dodecatriene,7,11-dimethyl- 3-methylene-,(Z)-	028973-97-9	95	0.22%
74	24.333	Decahydro-4a-methyl-methylene-7-(1-methylethenyl)-,[4aR-(4a.alpha.,7.alpha.,8a.beta.)]-	017066-67-0	99	0.15%
75	24.533	1H-Cycloprop[e]azulene,1a,2,3,4,4a,5,6,7b-octahydro-1,1,4,7-tetramethyl-,[1aR-(1a.alpha.,4.alpha.,4a.beta.,7b.alpha.)]-	000489-40-7	86	0.04%
76	24.688	Isobornyl propinonate	002756-56-1	38	0.05%
77	24.792	Naphthalene,1,2,4a,5,6,8a-hexahydro-4,7-dimethyl-1-(1-methylethyl)-	000483-75-0	97	0.01%
78	24.851	Naphthalene,1,2,3,,5,6,8a-hexahydro-4,7-dimethyl-1-(1-methylethyl)-,(1S-cis)-	000483-76-1	99	0.01%
79	24.904	Naphthalene,1,2,3,4-tetrahydro-1,6-dimethyl-4-(1-methylethyl)-,(1S-cis)-	000483-77-2	96	0.02%
80	25.715	Caryophyllene oxide	001139-30-6	90	0.05%
81	25.845	Hentriacontane	000630-04-6	38	0.01%
82	26.592	5.beta.,7.beta.H,10.alpha.-Eudesm-11-en-1.alpha.-ol	025826-85-1	52	0.02%
83	26.768	1-Propene,2-(3-methylphenyl)-1-phenyl-,(Z)-	000138-72-6	70	0.01%
84	28.239	Phthalic acid,2-ethoxyethyl octyl ester	1000322-87-6	50	0.01%
85	31.539	1,1,1,5,7,7,7-Heptamethyl-3,3-bis (trimethylsiloxy)tetrasiloxane	038147-00-1	32	0.04%

### Effect of EOs and Eucalyptol on APAP-Induced Hepatotoxicity

**Figure 3:**
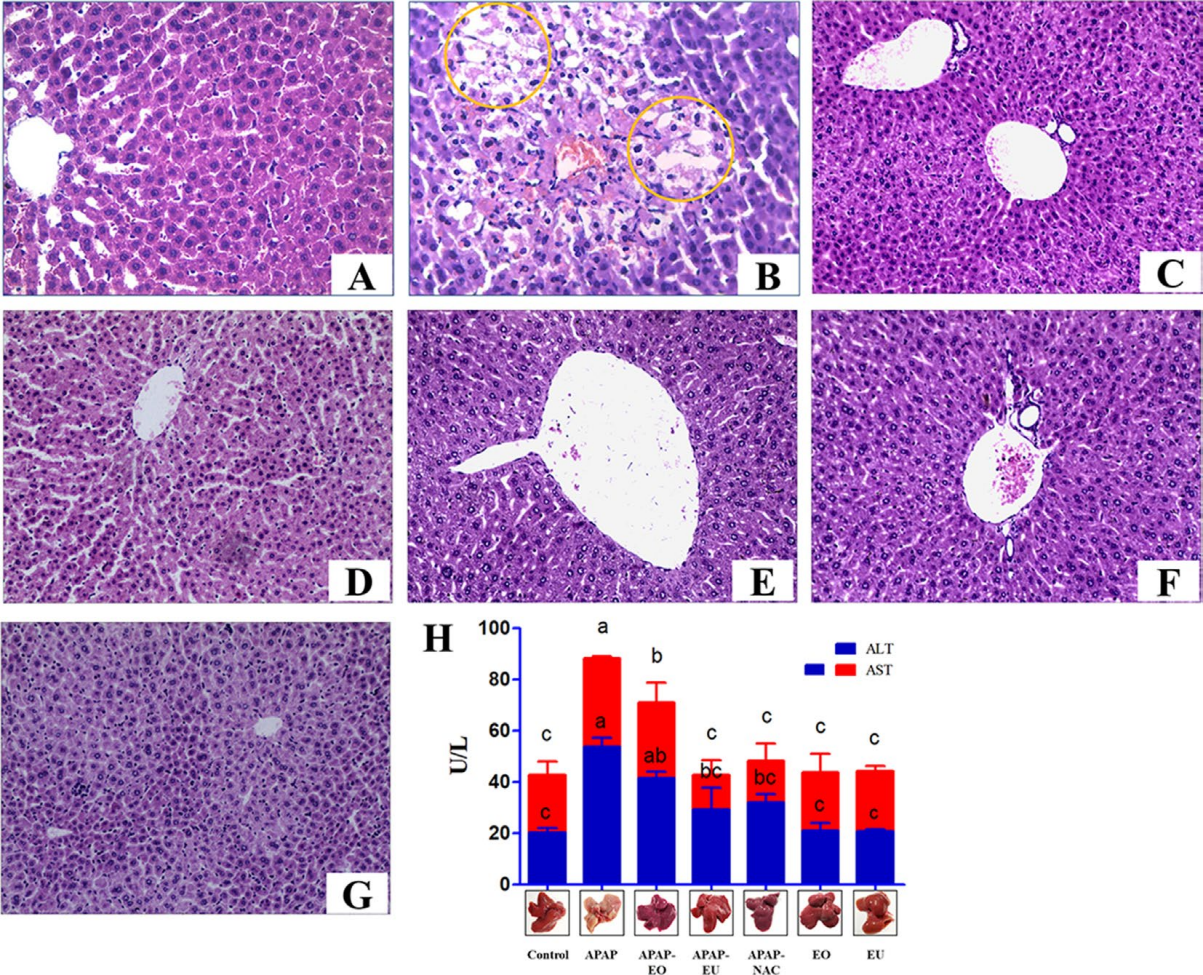
Effect of essential oil and eucalyptol on the acetaminophen (APAP)-induced liver changes and serum biomarkers of liver toxicity in mice. **(A–E)** The liver changes in mice. **(A)** Control group; **(B)** APAP treatment group, circle means cytoplasmic vacuolation; **(C)** APAP-EO treatment group; **(D)** APAP-EU treatment group; **(E)** APAP-NAC treatment group; **(F)** EO treatment group; **(G)** EU treatment group; **(H)** the activity of ALT and AST. *n* = 6, bars that do not share a common letter (a, b, c) were considered significantly different from each other (*p* < 0.05).

APAP-treated mice exhibited hepatocellular injury **(** **Figure 3B** **)** compared with control group (**Figure 1A**). More than half of the centrilobular hepatocytes were swollen with marked cytoplasmic vacuolation and condensed nuclei. The white spots were on the liver, while the EO and eucalyptol group did not show hepatotoxic effects (**Figures 3C–G**). ALT and AST levels were significantly increased by 2.67-fold and 2.06-fold in the APAP group, respectively, as compared to the control group **(** **Figure 3F** **)**. APAP-EO and APAP-EU decreased the ALT and AST activities compared with APAP-treated liver. The liver indexes were 4.43, 4.98, 4.48, 4.46, 4.42, 4.41, and 4.42 in the control-, APAP-, APAP-EO-, APAP-EU-, APAP-NAC-, EO, and EU group, respectively.

### Hepatic Antioxidant Effects of AV-EOs and EU on Oxidative Stress Biomarkers

**Figure 4:**
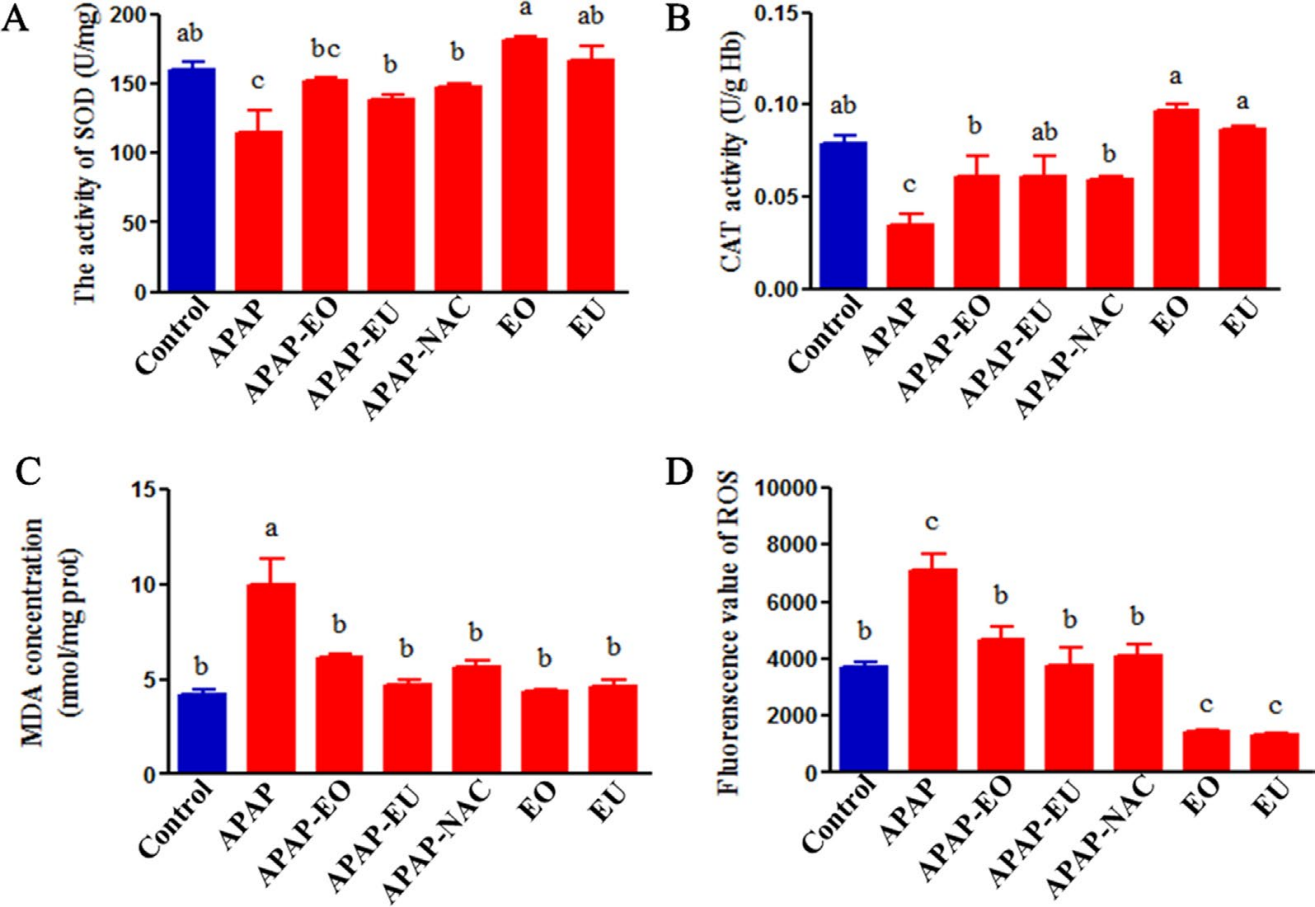
Effects of essential oil and eucalyptol on the APAP-induced oxidative stress parameters. ^a, b, c^ different letters indicate statistically different groups (*p* < 0.05).

To investigate the antioxidant effect of AV-EOs and EU, the levels of oxidative stress biomarkers were examined in mice **(** **Figure 4** **)**. APAP treatment significantly increased the content of MDA and ROS by 2.38- and 1.92-fold, respectively. APAP administration resulted in a significant decrease in the activities of SOD and CAT to 0.72- and 0.44-fold.

APAP-EO or EU co-administration reversed the decrease in OD and CAT and reduced the levels of ROS and MDA. Meanwhile, EOs and eucalyptol could eliminate ROS (*p* < 0.05).

### Effect of AV-EU on Oxidative Stress-Related Gene Expression

**Figure 5:**
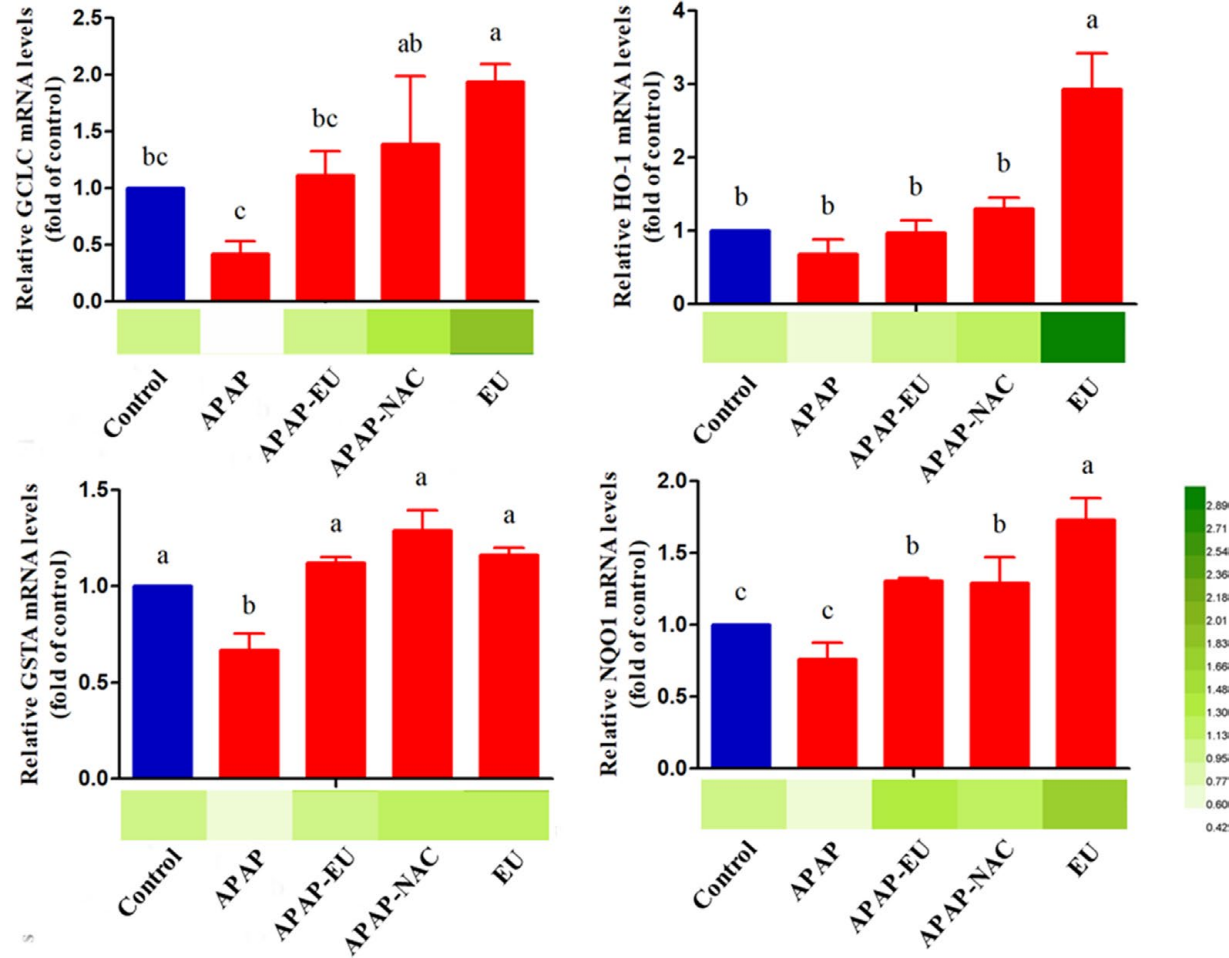
mRNA expression levels of oxidative stress-related genes . ^a, b, c, d^ different letters indicate statistically different groups (*p* < 0.05).

qPCR analysis indicated that APAP-EU co-treatment alleviated APAP-induced reduction of GCLC and GSTA. EU treatment significantly increased the levels of HO-1, NQO1, GCLC, and GSTA2, suggesting EU’s activities in antioxidant mediation **(** **Figure 5** **)**.

### Effect of AV-EU on APAP Metabolism

**Figure 6:**
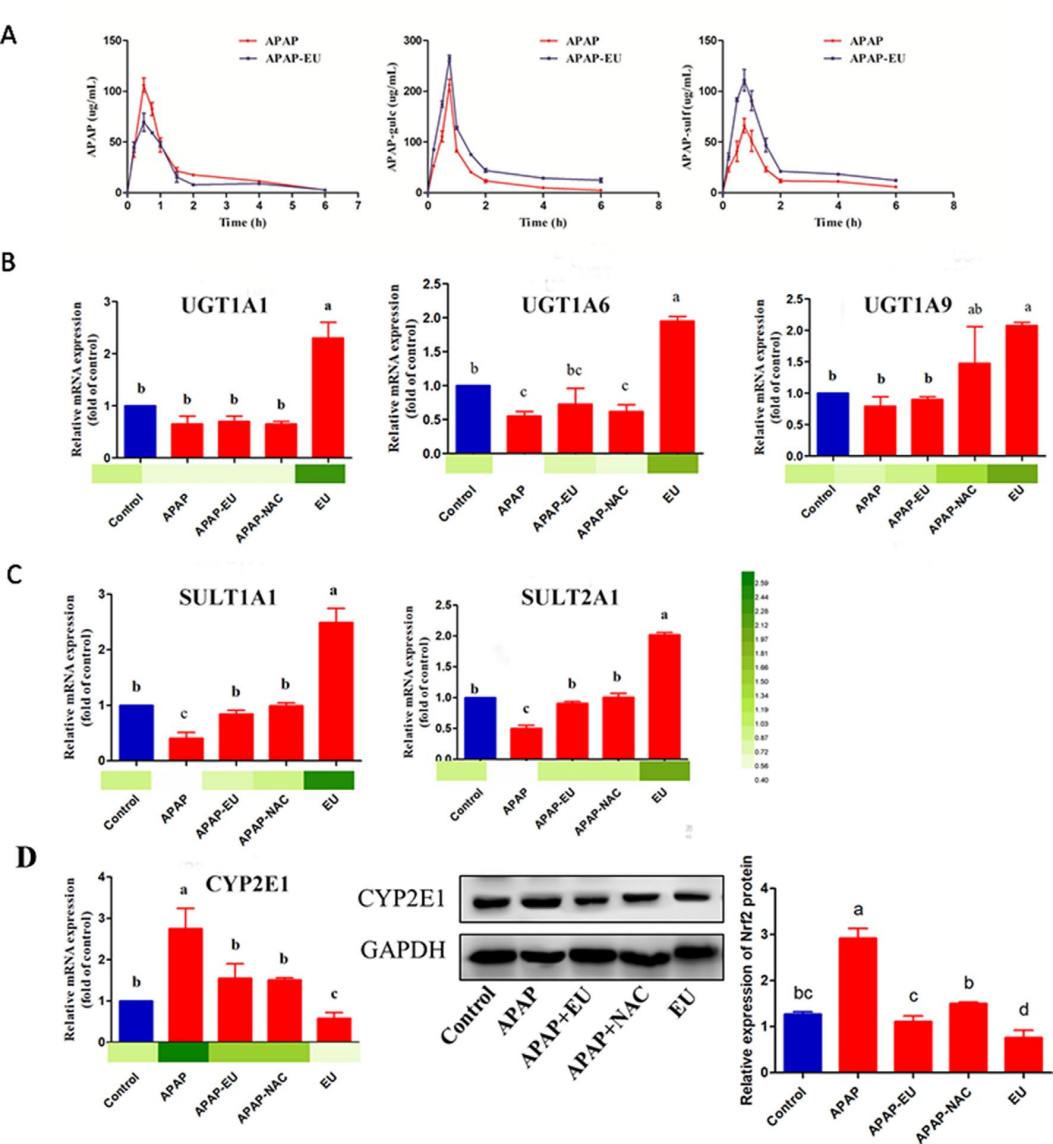
Effects of eucalyptol on the APAP metabolic disposition. APAP: APAP treatment group; APAP+EU: EU was intragastrically administrated after APAP treatment group; APAP+NAC: NAC was oral administration after APAP treatment group. EU: EU was intragastrically administrated. ^a, b, c^ different letters indicate statistically different groups (*p* < 0.05).

APAP and its major conjugates in plasma were analyzed using HPLC-MS/MS. The AUC of APAP in the APAP group was significantly higher (1.65 fold) than in APAP-EU treatment group, and APAP-EU significantly increased the levels of AUC of APAP-gulc and APAP-sulf in plasma **(** **Figure 6A** **)**.

The hepatic mRNA expressions of UGT1A1, UGT1A6, and UGT1A9 in the eucalyptol-treated group were 2.3-, 1.95-, and 2.07-fold higher than the control group **(** **Figure 6B** **)**, respectively. Compared with the control group, the mRNA expression of UGT1A6 significantly decreased 0.45-fold after APAP treatment. AV-EU post-treatment increased the mRNA levels of UGT1A6. Likewise, eucalyptol increased SULT1A1 and SULT2A1 mRNA levels by 2.5- and 2.0-fold, respectively **(** **Figure 6C** **)**. The expression of CYP2E1 was significantly increased (2.8-fold) after APAP treatment; the mRNA levels of CYP2E1 significantly increased after eucalyptol treatment **(** **Figure 6D** **)**.

### Effect of AV-EU on Nrf2 Expression

**Figure 7:**
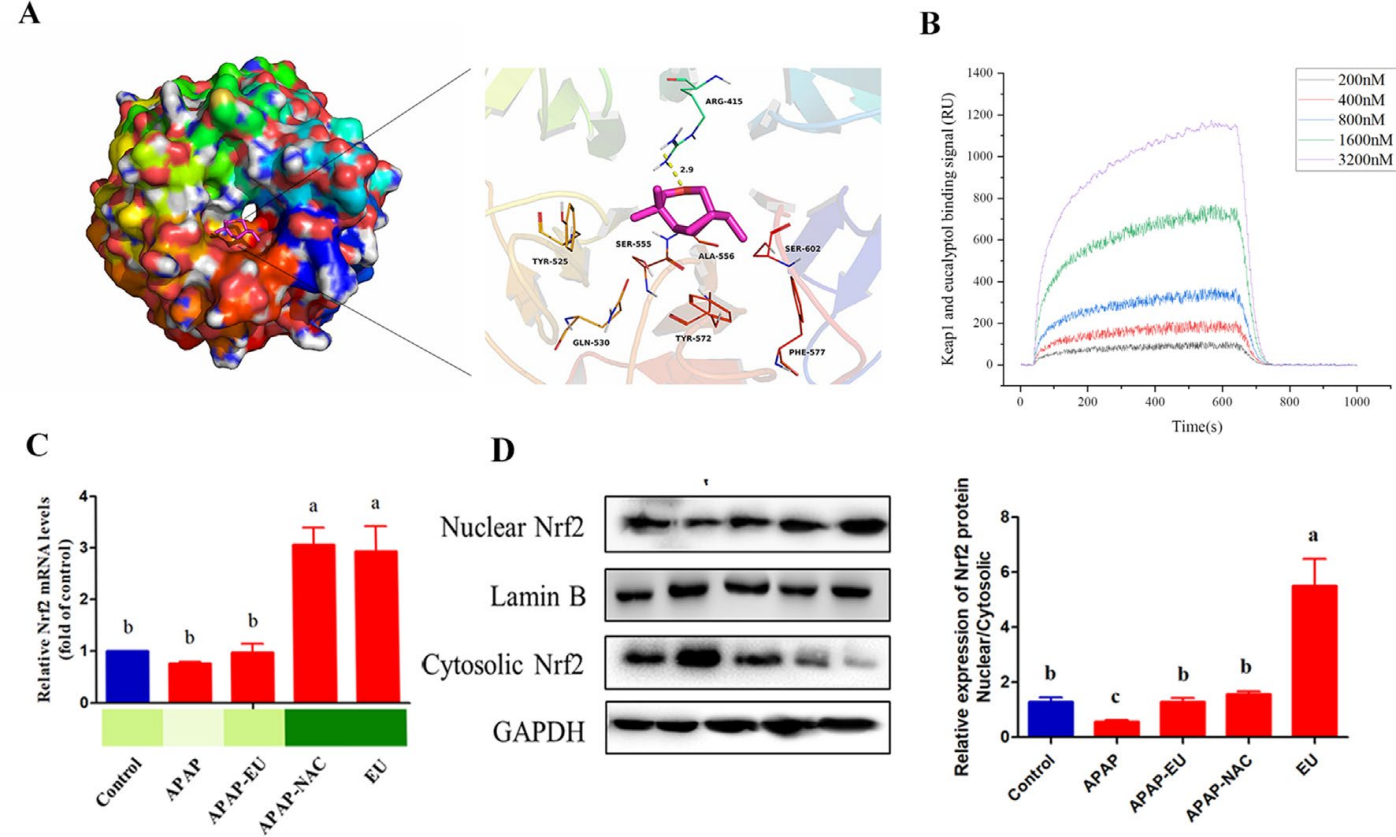
Effect of eucalyptol on nuclear factor erythroid 2-related factor 2 (Nrf2) expression. **(A)** eucalyptol docking with Keap1; **(B)** binding signal of Keap1 and eucalyptol (RU); **(C)** mRNA levels of Nrf2; **(D)** protein levels of Nrf2 in nuclear and cytosolic. ^a, b, c^ different letters indicate statistically different groups (*p* < 0.05).

The theoretical binding mode of the eucalyptol in the Nrf2 binding site of the Keap1 was illustrated in **Figure 7A**. Detailed analysis showed that the compound eucalyptol was positioned at the hydrophobic pocket, surrounded by the residues Tyr-525, Ala-556, Tyr-572, and Phe-577, forming a stable hydrophobic binding. Importantly, the “O” atom of eucalyptol formed the key hydrogen-bond interaction with the residue Arg-415, with a bond length of 2.9 Å. The hydrogen bond was the main interaction between eucalyptol and Keap1. According to the affinity measurement, eucalyptol showed a strong affinity (*K* *_D_* = 1.42 × 10^−5^) for Keap1 protein. The association rate constants *k* *_on_* and *k* *_off_* were 1.75 × 10^3^ and 2.49 × 10^−2^, respectively. Their binding curves during the test are shown in **Figure 7B**. These interactions helped to anchor eucalyptol in the Nrf2 binding site of Keap1. In addition, the estimated binding energy of eucalyptol is −5.5 kcal•mol^−1^, suggesting that eucalyptol is an inhibitor of the Keap1.

qPCR and WB analysis show that eucalyptol treatment increased Nrf2 mRNA **(** **Figures 7B, C** **)** and stimulated the nuclear translocation of Nrf-2 transcription factor. The ratio of protein expression of nuclear Nrf-2 and cytoplasmic Nrf-2 in eucalyptol was 5.8-fold higher than that of the control group **(** **Figure 7C** **)**.

## Discussion

AV is one of the famous *Artemisia* species compared to others such as *A. absinthium*, *A. nilagirica*, and *A. deserti*, (Kazemi et al., 2013; Pelkonen et al., 2013; Sati et al., 2013). AV-EOs have a high content of eucalyptol and Cis-β-Terpineol, which shown anti-inflammatory and antioxidant effects against various diseases, including respiratory disease, pancreatitis, colon damage, and non-alcoholic steatohepatitis (Murata et al., 2015; Seol and Kim, 2016). In our present study, treatment with AV-EOs and eucalyptol significantly attenuated APAP overdose (300 mg/kg) and induced the increase of serum aminotransferase and hepatic histopathological lesions **(** **Figure 3** **)**, suggesting that AV-EOs possess the ability to prevent APAP-induced hepatotoxicity.

Many studies on *Artemisia* did not result in any significant adverse effects in food/water consumption, body weight, mortality, hematology, serum biochemistry, organ weight, and histopathology (Wan et al., 2017; Yun et al., 2017); high-dose (752 mg/kg) usage of *Artemisia* may pose health based on mutagenesis and hepatotoxicity, suggesting that high-dose application of the extract in the treatment of serious disease is not recommended (Kalantari et al., 2013). *Artemisia abstinthium* may have neurotoxic; because of the major activity of thujone, it can inhibit the gammaaminobutyric acid A (GABAA) receptor causing excitation and convulsions in a dose-dependent manner (Pelkonen et al., 2013). In our study, AV-EOs do not contain thujone **(** **Table 2** **)**, and the dose of essential oil from *Artemisia vulgaris* was lower than the toxic dose. Our data show that dose of essential oil from Artemisia vulgaris (5%, 100 ml/kg) did not induce the liver injury. The liver index in the APAP group was significantly higher than that of the control group, while no significant difference was observed among APAP-EO _(AV)_, EO, and control group. Compared with compounds of EOs and the liver function indicators after EO treatment, we speculate that low dose of AV is safe.

APAP has non-toxic and toxic metabolic pathways in the liver. In the non-toxic pathway, APAP was glucuronidated and sulfated into APAP-gluc and APAP-sulf and excreted into blood and bile with the involvement of UGT and SULT family (Cao et al., 2017). In this study, the levels of UGT1A1, UGT1A6, UGT1A9, SULT1A1, and SULT2A1 were significantly increased after eucalyptol treatments, suggesting eucalyptol-enhanced APAP metabolism by the non-toxic pathway. The Nrf2 gene, with the consensus of TGAG/CNNNGC (N represents any base), is essential for inducing an increase in UGT and SULT family’s expression (Hayes and Dinkova-Kostova, 2014). Eucalyptol significantly increased the mRNA expression of Nrf2 **(** **Figure 7B** **)**. Eucalyptol can directly combine with Keap1 at site Arg-415, which was Nrf2-Keap1 binding site. Eucalyptol showed a strong affinity for Keap1 protein. The activated Nrf2 was transferred from cytoplasmic into nuclear. The expression of Nrf2 mRNA was also increased, resulting in an increase in nuclear/cytosolic relative expression under EO treatment **(** **Figure 7C** **)**. In addition, Nrf2 is a transcription factor that modulates endogenous antioxidant enzymes (Hou et al., 2018; Liu et al., 2018; Mahmoud et al., 2018). EOs stimulate Nrf2 activation. The activated Nrf2 binds to the antioxidant response element and further activates the transcription of gene encoding for antioxidants and detoxifications including heme oxygenase-1 (HO-1), NAD(P)H: quinone oxidoreductase-1 (NQO-1), and glutathione-synthesizing enzymes [glutamate-cysteine ligase catalytic subunit (GCLC)] (Hu et al., 2018). Our results suggest that EOs increased Nrf2 transfer from the cytoplasm to the nucleus, thereby leading to the transcriptional activation of antioxidant enzymes (HO-1, SOD, and CAT; Figs. 4 and 5) and phase II metabolic enzymes (UGT and SULT, **Figure 6**).

The second metabolism pathway of APAP was a toxic reaction. Overdosed APAP was transferred into NAPQI by CYP2E1 (Ganetsky et al., 2018), which undergoes chemical and enzymatic conjugation to GSH. The toxic pathway could lead to lipid peroxidation; antioxidant enzyme activities were reduced, and the levels of ROS were increased. Here, APAP increased MDA and ROS levels and decreased the activities of SOD and CAT, suggesting that APAP-induced hepatic dysfunction is caused by oxidative stress. The expression of CYP2E1 was significantly increased by APAP and decreased by eucalyptol treatment **(** **Figure 6** **)**. CYP2E1-deficient mice were resistant to the liver injury-induced APAP, while the transgenic mouse expressing human CYP2E1 was susceptible to the conversion of APAP to NAPQI. Our results show that eucalyptol inhibits CYP2E1 expression and attenuates liver injury. The pathway analysis study also reported the following active compounds: 2-Butanone,3-methyl-, 1-Octene, Benzaldehyde, Phenol,4-methyl-, Octanal, 1-Nonene, and Hexanal. These compounds represent the effective targets such as Aldo-keto reductase family 1 member C1, Alcohol dehydrogenase 1B, Cytochrome P450 2A6, and Cytochrome P450 1A2, which are involved in the metabolism of xenobiotics by cytochrome P450 and drug metabolism–cytochrome P450 pathway, respectively, which is related to CYP2E1 expression.

**Figure 8:**
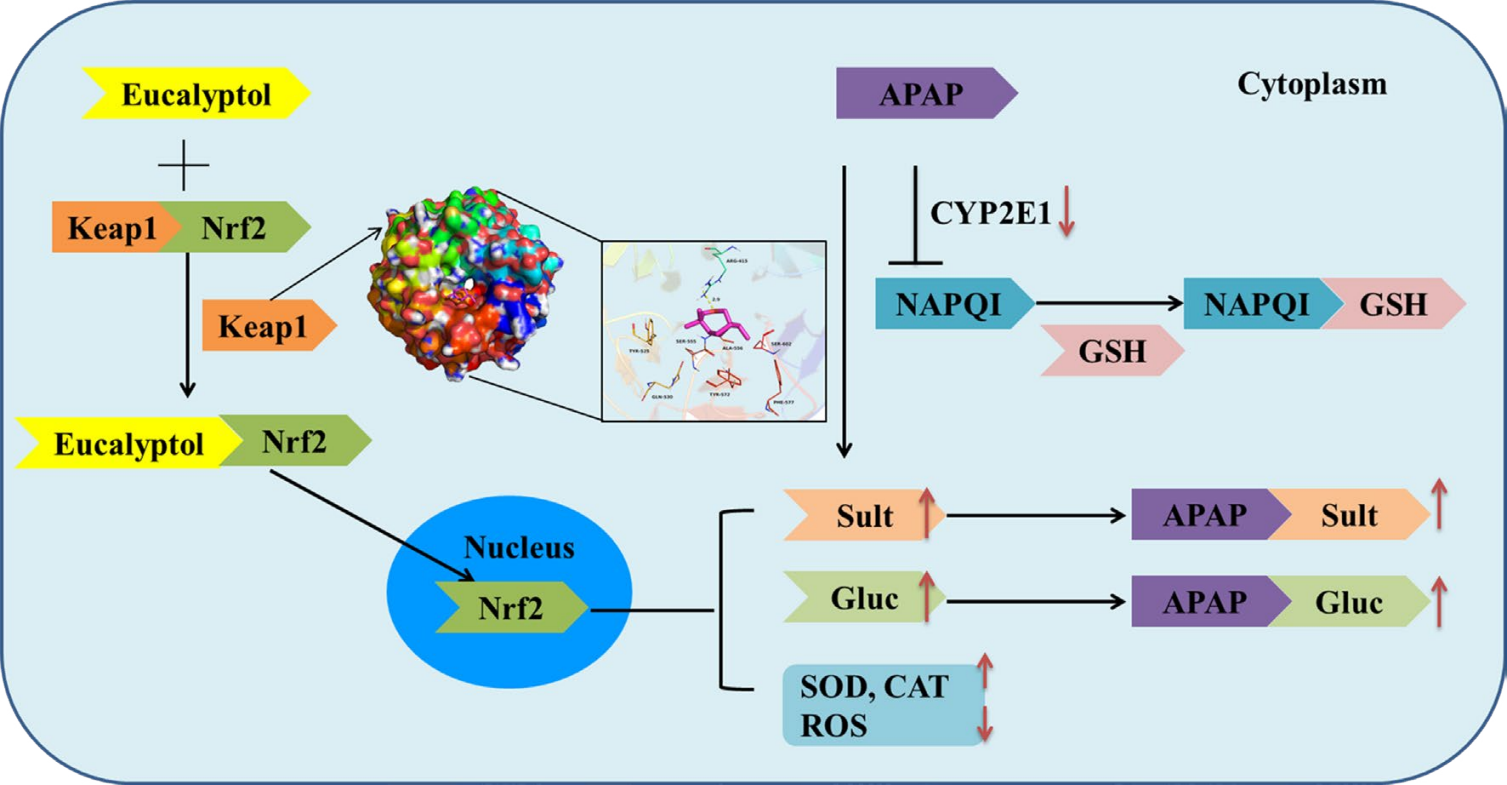
Mechanism by which eucalyptol protects APAP induced liver injury.

Collectively, AV-EOs can prevent APAP-induced liver injury through two pathways: down-regulation of CYP2E1 expression, which decreases plasma concentration of APAP into NAPQI, and the up-regulation of the expression of the detoxification pathway. The up-regulation pathway includes inhibitory binding with Keap1, which stimulates Nrf2 translocation from cytoplasm into mitochondria, activates Nrf2, and thus increases the activity of antioxidant enzymes (SOD, GSH, CAT, and GPx) and phase II enzymes (SULTs and UGTs) **(** **Figure 8** **)**, thereby decreasing APAP plasma concentration and accelerating APAP harmless metabolism.

## Data Availability

All datasets generated for this study are included in the manuscript and the supplementary files.

## Ethics Statement

The mice were obtained from the animal center of Zhengzhou University (Zhengzhou, China). The experimental protocol was reviewed and approved by the Ethics Committee of the Institute of Modern Biotechnology for the Use of Laboratory Animals.

## Author Contributions

ZJ and XG designed the study, performed the research, analyzed data, and wrote the paper; KZ, GS, BC, QZ, and SZ performed animal research and analyzed data. GK and XZ designed research and organized the discussion.

## Funding

This work was supported by the Key Projects of Universities in Henan (19B180001), Science, Technology Innovation Talents in Universities of Henan Province (18HASTIT035), and National Key R&D Program of China (2017YFD0501003).

## Conflict of Interest Statement

The authors declare that the research was conducted in the absence of any commercial or financial relationships that could be construed as a potential conflict of interest.
