# Supplementary material for: The Essential Oils and Eucalyptol From Artemisia vulgaris L. Prevent Acetaminophen-Induced Liver Injury by Activating Nrf2–Keap1 and Enhancing APAP Clearance Through Non-Toxic Metabolic Pathway
Source: Front Pharmacol. 2019 Jul 25;10:782. doi: 10.3389/fphar.2019.00782 (PMC6669816; doi:10.3389/fphar.2019.00782)
Supplement: Supplementary file 2 [file Table_2.docx]

Supplementary Table 2 Inter-species variations of ITS2 sequences of Artemisia species

|  | Sample 1 | Sample 2 | *Artemisia annua* | *Artemisia argyi* | *Artemisia lavandulifolia* | *Artemisia vulgaris* |
| --- | --- | --- | --- | --- | --- | --- |
| Sample 1 | - | - | - | - | - | - |
| Sample 2 | 0.009 | - | - | - | - | - |
| *Artemisia annua* | 0.054 | 0.051 | - | - | - | - |
| *Artemisia argyi* | 0.005 | 0.011 | 0.049 | - | - | - |
| *Artemisia lavandulifolia* | 0.024 | 0.023 | 0.033 | 0.019 | - | - |
| *Artemisia vulgaris* | 0.009 | 0.003 | 0.048 | 0.009 | 0.019 | - |
